# Patient attitudes toward HPV self-sampling and community health worker-delivered cervical cancer screening services in an underserved area

**DOI:** 10.1017/cts.2025.10172

**Published:** 2025-11-17

**Authors:** Mrithula Suresh Babu, Monica L. Kasting, Natalia M. Rodriguez

**Affiliations:** 1 Department of Public Health, College of Health and Human Sciences, Purdue University, West Lafayette, IN, USA; 2 Cancer Prevention and Control Program, Indiana University Simon Comprehensive Cancer Center, Indianapolis, IN, USA

**Keywords:** Self-sampling, cervical cancer, cancer screening, community health workers, human papillomavirus

## Abstract

**Introduction:**

In Indiana, Black women had 12% higher cervical cancer incidence, and 21% higher mortality compared to White women during the years 2008–2017. Human Papillomavirus (HPV) self-sampling delivered by community health workers (CHW) has demonstrated potential to increase cervical cancer screening rates among at-risk populations. This study aims to understand patient attitudes toward HPV self-sampling and CHW-delivered screening.

**Methods:**

We conducted a cross-sectional online survey among patients from three Planned Parenthood clinics in Lake County, IN, which has one of the highest cervical cancer mortality rates in Indiana. Patients’ willingness to self-sample and to receive health education and services related to cervical cancer screening from CHWs was analyzed using logistic regression.

**Results:**

In the final sample (*N* = 140), the largest proportions of respondents were below age 30 (54.3%), aware of cervical cancer (78.6%), up to date on cervical cancer screening (84.3%), had no prior self-sampling experience (53.6%) and were aware of CHWs (52.1%). Multivariable logistic regression analyses showed Black patients had higher odds of being willing to: self-sample at home (aOR = 3.749, 95% CI = 1.619–8.681), receive health education from CHWs (aOR = 4.952, 95% CI = 2.124–11.548), and receive health services from CHWs (aOR = 3.305, 95% CI = 1.479–7.388) compared to White patients.

**Conclusion:**

Our results showed Black patients had higher willingness for HPV self-sampling and CHW delivery of cervical cancer screening services. Study findings can be used to inform future CHW-led interventions for outreach, education, and delivery of self-sampling interventions to increase cervical cancer screening rates among Black women in underserved areas.

## Introduction

In the United States, there are significant racial disparities in cervical cancer incidence and mortality, a disease that can be prevented and cured through regular screening. Prior evidence show that Black women have higher cervical cancer incidence and mortality rates compared to non-Hispanic White women [1–3]. In Indiana, Black women had 12 percent higher cervical cancer incidence and 21 percent higher mortality compared to White women during the years 2008 to 2017 [4]. Lake County, Indiana, has the second largest Black (24.9%) population in the state [5]. From 2018–2022, the cervical cancer mortality rate was 3.1 per 100,000 in Lake County when compared to the state average of 2.8 per 100,000 women per year [6] and national average of 2.2 per 100,000 women per year [7].

Early detection of cervical cancer plays a vital role because Human Papillomavirus (HPV) infection, the virus that causes nearly all cervical cancers, is usually asymptomatic in nature and precancerous lesions have a slow progression [8–10]. In 2018, the United States Preventive Services Task Force (USPSTF) guidelines were updated to include primary HPV testing every 5 years for women between the ages of 30 and 65 years, Pap testing every 3 years or co testing every 5 years [11].

The Centers for Disease Control and Prevention (CDC) established the National Breast and Cervical Cancer Early Detection Program (NBCCEDP) through the Breast and Cervical Cancer Mortality Prevention Act to help provide breast and cervical cancer screening to uninsured, low-income women. The NBCCEDP program has also partnered with organizations such as Young Women’s Christian Association (YWCA) and American Cancer Society to improve screening rates among underserved groups [12]. The effects of the NBCCEDP program on improving healthcare outcomes for low income, uninsured women through cervical cancer screening have been studied. The findings showed that mortality rates have reduced for women participating in the program [13]. In 1996, the Breast and Cervical Cancer Program (BCCP) was established in Indiana as a part of the NBCCEDP to improve screening among low-income and uninsured women [14]. Despite these efforts, the cervical cancer screening rate in Indiana was 76% in 2020, below the national average [15]. This highlights the need for implementing novel screening strategies to increase cervical cancer screening rates in Indiana.

A systematic review demonstrated that some of the barriers to cervical cancer screening faced by minority women in the United States are low knowledge, individual perceptions of risk and screening methods, feelings of discomfort and embarrassment, language barriers, and health insurance [16]. Other study findings showed that lack of access to care and lack of health insurance were the major predictors in the underutilization of the cervical cancer screening services [17] and in Indiana, lack of insurance was associated with later stages of cervical cancer diagnosis [18]. The 2021 President’s Cancer Panel report highlighted how barriers to cervical cancer screening could be mitigated by using novel screening strategies such as HPV self-sampling and community health worker (CHW) navigation [19]. First, HPV self-sampling involves the collection of vaginal material by the patient herself using swabs and it does not involve a pelvic examination by a provider [20]. HPV self-sampling could increase cervical cancer screening rates among at-risk populations [21–23] by mitigating some of the barriers related to in-clinic cervical cancer screening such as discomfort and pain, embarrassment, privacy concerns, and challenges related to taking time off work [24–26]. A mixed-methods study focused on factors related to acceptability of HPV testing showed that higher knowledge, perceived susceptibility, and subjective norms influence testing uptake, which is informative for researchers designing interventions to improve screening rates [27]. Second, CHWs usually have shared demographic characteristics and built rapport with their community and offer a broad range of health services based on the needs of the community [28]. Therefore, CHW-based interventions have also proven effective in addressing cervical cancer knowledge and increasing screening uptake among minority women [29–33]. Another study showed that engaging CHWs is cost-effective and was associated with net healthcare savings [34,35].

Given the disparities experienced by the minority populations in Lake County, Indiana, and the promise shown by engaging CHWs in self-sampling, we partnered with Planned Parenthood clinics to engage with their patients directly. Planned Parenthood clinics provide preventive healthcare and women’s cancer screenings in medically underserved communities, and at the time of the study had 3 open clinics in the Lake County area. The objective of this community-engaged study was to understand patient willingness to adopt innovative cervical cancer screening strategies such as HPV self-sampling and CHW navigation to address common barriers to screening and improve linkage to care in the community.

## Materials and methods

### Study design

In our community-engaged research study, we conducted a cross-sectional survey from May through July 2022 among patients ages 21–65 from three Planned Parenthood clinics – Gary, Hammond, and Merrillville – in Lake County, Indiana. This study was approved under expedited review by the Purdue University Institutional Review Board (IRB 2021-1385).

### Recruitment

Planned Parenthood sent a preapproved message to all eligible Lake County patients with information about the study and survey link through their patient portal. Recruitment flyers were also posted and handed out in waiting rooms of the three Lake County clinics. The participants had the option of taking the survey online using the QR code or in-person at the clinic with the assistance of a study team member. The eligibility criteria included: patients assigned female at birth, aged 21 to 65 years old, who visited any of the Lake County Planned Parenthood clinics from 2018–2021.

### Data collection

A total of 227 patients completed the survey. Patients were excluded from the final analyses if they had not been to a Planned Parenthood in the past four years (*n* = 22), were assigned male at birth (*n* = 1), underwent hysterectomy (*n* = 9), had unknown insurance status (*n* = 19) or were of a racial/ethnic group other than non-Hispanic White and non-Hispanic Black groups (*n* = 36). The remaining sample (*N* = 140) had completed all the questions related to the three outcome measures and so responses were not excluded based on missing data. Therefore, the final analytic sample included 140 patients.

### Measures

The survey included 68 items and took an average of 11 minutes to complete. Based on the frequencies of some of the categorical variables, some responses were collapsed based on responses and shared characteristics. For example, education was recoded into three categories – “less than 8^th^ grade” and “high school/GED” were combined into “high school or less,” “post high school training other than college” and “some college” were combined into “some college,” and “college graduate” and “postgraduate” were combined into “college graduate.” Moreover, some variables were collapsed due to low frequencies of responses. An example of this is age, which originally had more than 10 categories and most of the cell counts were less than 5. The full list of measures and response options are as follows: demographic characteristics included age (dichotomous – 30 and under, 31 and above), household income (three categories – under $20,000, $20,000–$40,000, and $40,000 and above), insurance status (three categories- private insurance, Medicaid, Medicare and uninsured/self-pay), marital status (three categories – married, widowed/divorced/separated, and never been married), race (dichotomous – non-Hispanic White and non-Hispanic Black), educational attainment (three categories – high school or less, some college and college graduate), and employment status (dichotomous – employed and unemployed).

In addition, there were questions related to cervical cancer knowledge and awareness, cervical cancer screening status, risk perception, CHW awareness, and willingness to receive education and cervical cancer screening services from a CHW. Cervical cancer knowledge score was calculated as a continuous variable using a sum score from seven questions adapted from previously published research articles [36–40]. Five of the knowledge questions were multiple choice questions and the remaining two questions had multiple response items. The correct responses were recoded as 1 and the other responses were recoded as 0, before creating the total knowledge score on a scale of 0–7 for each participant. Awareness of cervical cancer was recoded as a dichotomous variable (yes/no). Participants were asked about their cervical cancer screening status, and the variable was recoded as a dichotomous variable (up to date on screening [based on 2018 USPSTF guidelines]/unscreened or overdue) based on whether they have had prior screening and the time period since their last screening exam. Risk perception was calculated based on the participants’ assessment of their risk of getting cervical cancer compared to other women from the same age group and their responses were recoded as “Higher” or “Same or Lower.” Finally, participants were asked about their awareness of CHWs and whether they received services from CHWs. These two variables were recoded as dichotomous variables (yes/no).

### Outcomes

The three main outcomes were: participants’ willingness to: (1) self-sample for cervical cancer, (2) receive health education from CHWs, and (3) receive services related to cervical cancer screening from CHWs. These were assessed with three different survey questions. They were asked to rate their agreement to the statement “*I would be willing to take my own vaginal sample at home with written instructions and send it to my doctor (via mail or dropping off at the clinic) to test for cervical cancer.*” with the Likert scale options being 1-Strongly agree to 5-Strongly disagree. Similarly, the participants were asked to respond to statements “*I would be willing to receive health education about cervical cancer delivered by a community health worker (CHW)*.” and *“I would be willing to receive other services related to cervical cancer screening from a community health worker (CHW). For example, verbal instructions on how to take my own vaginal swab, help sending my sample back to a clinic, help scheduling an appointment with my doctor.”* with a similar Likert scale. Because the aim of this study was to examine willingness to receive self-sampling, receive health education and health services from CHWs, we decided to dichotomize into those who indicated they were willing, by selecting “strongly agree” or “agree” and compare it to all other responses. Therefore, all the three categorical variables were recoded to binary outcome variables with the “Strongly agree” and “Agree” groups combined as a single group and similarly, the “Neither agree nor disagree,” “Disagree” and “Strongly disagree” groups were combined in a separate group.

### Statistical analysis

Descriptive statistics were calculated for all the survey questions. We examined patients’ willingness to self-sample, receive health education, and services related to cervical cancer screening from CHWs using logistic regression analyses. First, we examined each variable’s individual association with the three outcomes using binary logistic regression analyses. Finally, we conducted multivariable regression analyses to examine associations between demographic characteristics, patients’ knowledge, willingness to self-sample at home and willingness to receive health education and cervical cancer-related screening services from CHWs. Because we wanted to analyze the patients’ acceptability of innovative screening strategies, we included all the demographic characteristics we collected in order to identify any predictors for willingness to self-sample, receive health education and health services from CHWs. Data analyses were carried out in SPSS version 29.

## Results

In the final sample (*N* = 140), the largest proportions of respondents were below the age of 30 (54.3%), had a household income of $40,000 and above (39.3%), had private insurance (45.7%), had never been married (69.3%), had graduated from college (48.6%) and were employed (73.6%). The majority of the sample were aware of cervical cancer (78.6%), were up to date on cervical cancer screening (84.3%), had a lower perception of their risk of cervical cancer compared to other women (74.3%), did not have prior self-sampling experience (53.6%) and were aware of CHWs (52.1%). Of the survey respondents who were aware of CHWs, most (64.2%) had not previously received any services from CHWs. Additional participant characteristics are described in Table 1. 45% (*n* = 63) of the respondents had visited the Hammond Health Center, 42% (*n* = 59) had visited the Merrillville Health Center and 18% (*n* = 18) had visited the Gary Health Center. There were no significant differences between participants at each clinic by sociodemographic characteristics or any of the three outcome variables. Therefore, participants from each of the separate clinics were all analyzed together (Supplementary Table 1).

**Table 1.** Demographic characteristics, cervical cancer knowledge, and awareness of CHWs

Variable	*N* (%)
**Age**	
30 and under	76 (54.3)
31 and above	64 (45.7)
**Household income**	
Under $20,000	32 (22.9)
$20,000–$40,000	53 (37.9)
$40,000 and above	55 (39.3)
**Insurance status**	
Private insurance	64 (45.7)
Medicaid	62 (44.3)
Medicare	6 (4.3)
Uninsured/self-pay	8 (5.7)
**Race/ethnicity**	
Non-Hispanic White	65 (46.4)
Non-Hispanic Black	75 (53.6)
**Marital status**	
Married	24 (17.1)
Widowed/divorced/separated	19 (13.6)
Never been married	97 (69.3)
**Education**	
High school or less	14 (10)
Some college	58 (41.4)
College graduate	68 (48.6)
**Occupation**	
Employed	103 (73.6)
Unemployed	37 (26.4)
**Awareness of cervical cancer**	
Yes	110 (78.6)
No/I don’t know	30 (21.4)
**Overdue for cervical cancer screening**	
Not screened/overdue	22 (15.7)
Screened	118 (84.3)
**Risk perception**	
Same or lower	104 (74.3)
Higher	36 (25.7)
**Prior self-sampling**	
Yes	65 (46.4)
No	75 (53.6)
**Awareness of CHWs**	
Yes	73 (52.1)
No	67 (47.9)
**Received services from CHWs**	
Yes	24 (35.8)
No	43 (64.2)
**Willingness to self-sample**	
Agree	82 (58.6)
Disagree	58 (41.4)
**Willingness to receive health education from CHWs**	
Agree	83 (59.3)
Disagree	57 (40.7)
**Willingness to receive services related to cervical cancer screening from CHWs**	
Agree	89 (63.6)
Disagree	51 (36.4)

The bivariate logistic regression analyses showed that non-Hispanic Black patients had higher odds of being willing to self-sample at home (Odds ratio, OR = 2.638, 95% Confidence interval, CI = 1.321–5.269) compared to non-Hispanic White patients. The multivariable logistic regression analyses also showed that non-Hispanic Black patients had higher odds of being willing to self-sample at home (aOR = 3.749, 95% CI = 1.619–8.681) compared to non-Hispanic White patients. Patients with higher knowledge score (aOR = 1.245, 95% CI = 1.009–1.535) and patients who were overdue for screening (aOR = 3.674, 95% CI = 1.077–12.538) had higher odds of being willing to self-sample at home (Table 2).

**Table 2.** Self-sampling results using logistic regression analyses

	Bivariate analyses		Multivariable analyses	
	**OR**	**95% CI**	**OR**	**95% CI**
**Age**	0.839	0.427–1.647	0.642	0.283–1.460
**Marital status**				
Married	Ref.		Ref.	
Widowed/divorced/ separated	0.571	0.154–2.123	0.685	0.155–3.019
Never been married	0.385	0.141–1.054	0.289*	0.092–0.911
**Education**				
High school or less	Ref.		Ref.	
Some college	2.739	0.814–9.215	3.095	0.707–13.556
College graduate	2.908	0.878-9.631	3.906	0.884–17.254
**Race/ethnicity**				
Non-Hispanic White	Ref.		Ref.	
Non-Hispanic Black	2.638**	1.321–5.269	3.749**	1.619–8.681
**Income**				
$40,000 and above	Ref.		Ref.	
Under $20,000	1.111	0.453–2.723	1.312	0.329–5.236
$20,000–$40,000	0.806	0.375–1.729	0.884	0.303–2.582
**Insurance**				
Private insurance	Ref.		Ref.	
Medicaid	1.077	0.532–2.182	1.306	0.454–3.755
Medicare	3.889	0.430–35.206	4.519	0.244–83.788
Uninsured/self-pay	1.296	0.285–5.892	3.513	0.523–23.598
**Employed**	1.285	0.603–2.741	1.803	0.682–4.766
**Knowledge score**	1.100	0.929–1.304	1.245*	1.009–1.535
**Overdue for screening**	2.772	0.959–8.010	3.674*	1.077–12.538
**Prior self-sampling**	0.554	0.279–1.099	0.774	0.343–1.747
**Risk perception**	1.181	0.549–2.538	1.254	0.508–3.094

* Significance at the 0.05 level. **Significance at the 0.01 level. ***Significance at the 0.001 level.

The bivariate logistic regression analyses showed that non-Hispanic Black patients had higher odds of being willing to receive health education from CHWs (OR = 3.192, 95% CI = 1.582–6.443) compared to non-Hispanic White patients. The multivariable logistic regression analyses also showed that non-Hispanic Black patients had higher odds of being willing to receive health education from CHWs (aOR = 4.952, 95% CI = 2.124–11.548) compared to non-Hispanic White patients (Table 3).

**Table 3.** CHW health education results using logistic regression analyses

	Bivariate analyses		Multivariable analyses	
	**OR**	**95% CI**	**OR**	**95% CI**
**Age**	0.704	0.357–1.387	0.575	0.249–1.329
**Marital status**				
Married	Ref.		Ref.	
Widowed/divorced/ separated	0.706	0.196–2.544	0.859	0.187–3.951
Never been married	0.517	0.197–1.360	0.523	0.170–1.610
**Education**				
High school or less	Ref.		Ref.	
Some college	1.231	0.383–3.960	1.356	0.313–5.868
College graduate	1.833	0.575–5.847	3.015	0.688–13.218
**Race/ethnicity**				
Non-Hispanic White	Ref.		Ref.	
Non-Hispanic Black	3.192**	1.582–6.443	4.952***	2.124–11.548
**Income**				
$40,000 and above	Ref.		Ref.	
Under $20,000	2.291	0.900–5.836	3.367	0.818–13.869
$20,000–$40,000	1.263	0.590–2.703	1.606	0.544–4.740
**Insurance**				
Private insurance	Ref.		Ref.	
Medicaid	1.312	0.645–2.667	0.555	0.180–1.709
Medicare	0.829	0.155–4.420	0.265	0.032–2.192
Uninsured/Self-pay	5.800	0.674–49.908	7.883	0.744–83.497
**Employed**	0.441	0.194–1.005	0.412	0.145–1.166
**Knowledge score**	0.908	0.765–1.078	0.934	0.754–1.157
**Overdue for screening**	1.576	0.598–4.151	1.910	0.579–6.303
**Prior self-sampling**	0.585	0.295–1.160	0.578	0.248–1.347
**Risk perception**	1.054	0.488–2.277	0.820	0.321–2.090

* Significance at the 0.05 level. **Significance at the 0.01 level. ***Significance at the 0.001 level.

The bivariate logistic regression analyses showed that non-Hispanic Black patients had higher odds of being willing to receive cervical cancer-related health services from CHWs (OR = 2.858, 95% CI = 1.402–5.826) compared to non-Hispanic White patients. The multivariable logistic regression analyses also showed that non-Hispanic Black patients had higher odds of being willing receive health services from CHWs (aOR = 3.305, 95% CI = 1.479–7.388) compared to non-Hispanic White patients (Table 4).

**Table 4.** CHW health services results using logistic regression analyses

	Bivariate analyses		Multivariable analyses	
	**OR**	**95% CI**	**OR**	**95% CI**
**Age**	0.918	0.460–1.832	1.096	0.491–2.447
**Marital status**				
Married	Ref.		Ref.	
Widowed/divorced/ separated	0.237*	0.062–0.900	0.183*	0.040–0.835
Never been married	0.446	0.153–1.297	0.417	0.125–1.395
**Education**				
High school or less	Ref.		Ref.	
Some college	1.636	0.506–5.295	1.713	0.448–6.547
College graduate	2.091	0.653–6.699	2.352	0.616–8.975
**Race/ethnicity**				
Non-Hispanic White	Ref.		Ref.	
Non-Hispanic Black	2.858**	1.402–5.826	3.305**	1.479–7.388
**Income**				
$40,000 and above	Ref.		Ref.	
Under $20,000	1.091	0.438–2.719	1.436	0.377–5.469
$20,000–$40,000	0.943	0.432–2.059	1.211	0.422–3.470
**Insurance**				
Private insurance	Ref.		Ref.	
Medicaid	0.951	0.461–1.965	0.706	0.241–2.066
Medicare	1.122	0.191–6.603	0.635	0.080–5.015
Uninsured/self-pay	0.935	0.205–4.274	1.351	0.234–7.801
**Employed**	1.269	0.587–2.744	1.091	0.421–2.830
**Knowledge score**	0.891	0.748–1.063	0.890	0.725–1.093
**Overdue for screening**	2.172	0.750–6.292	2.627	0.786–8.783
**Prior self-sampling**	0.716	0.357–1.435	0.750	0.329–1.710
**Risk perception**	0.833	0.375–1.852	0.677	0.267–1.715

* Significance at the 0.05 level. **Significance at the 0.01 level. ***Significance at the 0.001 level.

## Discussion

The study findings showed that non-Hispanic Black patients demonstrated higher willingness for self-sampling at home and CHW delivery of health education and services related to cervical cancer screening, when compared to non-Hispanic White patients. Overall, patients in the sample showed a high awareness of cervical cancer and CHWs, and the majority of the patients were up to date with their cervical cancer screening. These findings must be interpreted in the context that the sample is entirely comprising of women who are already in care, as patients of Planned Parenthood clinics in Lake County, IN.

Consistent with our findings, Black women in the Mississippi Delta showed higher uptake for HPV self-sampling and those study findings also demonstrated the cost-effectiveness of HPV self-sampling [41]. Similarly, a conjoint analysis showed that Black women found HPV self-sampling to be acceptable [42]. Furthermore, women from other countries such as Japan, Brazil have also shown a higher acceptability toward self-sampling approaches when compared to physician conducted sampling for cervical cancer screening [43,44].

Previous study findings show that HPV self-sampling decreases screening costs when compared to HPV testing in a clinic [45,46]. The cost-effectiveness of HPV self-sampling is particularly important in consideration of low-resource and low-income settings such as Lake County, Indiana. In addition, self-sampling could help overcome barriers related to screening such as privacy concerns, taking time off from work and long wait times for screening appointments for women [47]. Moreover, developing educational tools regarding the proper collection, storage, and transportation of the sample is important to ensure the accuracy of the results [48].

CHW-led interventions have been proven to be successful in improving colorectal cancer screening rates in racial and ethnic minority groups [49]. Another study evaluating CHW-led interventions related to colorectal cancer screening showed how facilitating access to care by linking them to a clinic, is an important factor in improving health outcomes [50]. Similarly, for cervical cancer, CHW-led interventions were effective in improving health literacy related to breast and cervical cancer screening [30,32]. Therefore, CHW-led interventions could be implemented to increase cervical cancer screening rates through self-sampling.

This study is among the first to focus on medically underserved counties in Indiana such as Lake County and highlight novel screening strategies such as self-sampling to address the low cervical cancer screening rates. However, it does have several limitations, and the results should be interpreted considering these limitations. First, because this study was conducted using clinic patients, the findings may not be generalizable to the general population and future work should expand to reach broader community members who may not yet be patients. There is potential selection bias as the respondents were recruited in the three clinics during their doctor’s visit. Additionally, other racial and ethnic minority groups have not been included in the data analysis due to low survey response rate. Therefore, we focused on participants who identified as Black and White, which limits the generalizability of our findings to other at-risk groups such as Hispanic, Native American or American Indian, and Asian or Pacific Islander groups. Measures such as country of origin, immigration status, family history, and other co-morbidities were not assessed in the survey, and this limited our ability to account for these differences in our population. Finally, the data analysis is based on self-reported data from the patient surveys and response bias could impact the selection of responses. The dichotomization of the outcome variables also leads to bias and loss of statistical power in the models. Future studies will address these limitations and expand on the partnership with Planned Parenthood to pilot self-sampling interventions and potentially scale-up throughout the state of Indiana.

## Conclusion

Study findings show that there is a higher willingness toward HPV self-sampling and CHW delivered services among Black patients in Planned Parenthood clinics in Lake County, IN. These findings highlight the fact that novel screening strategies such as patient self-sampling and CHW delivered services can be leveraged to deliver context-specific interventions in medically underserved communities such as Lake County, IN.

## Supporting information

10.1017/cts.2025.10172.sm001 Babu et al. supplementary material Babu et al. supplementary material

## Supplementary material

The supplementary material for this article can be found at https://doi.org/10.1017/cts.2025.10172.
