# Supplementary material for: Patient attitudes toward HPV self-sampling and community health worker-delivered cervical cancer screening services in an underserved area
Source: J Clin Transl Sci. 2025 Nov 17;9(1):e270. doi: 10.1017/cts.2025.10172 (PMC12722056; doi:10.1017/cts.2025.10172)
Supplement: Babu et al. supplementary material [file S2059866125101726sup001.docx]

**Supplementary Table 1 – Analysis of the outcomes across clinic sites**

| **Outcomes, n (%)** | | **Clinic sites** | | | **p-value** |
| --- | --- | --- | --- | --- | --- |
|  |  | **Hammond Health Center** | **Merrillville Health Center** | **Gary Health Center** |  |
| **Self-sampling** | **Agree** | 40 (63.5) | 39 (66.1) | 15 (83.3) | .280 |
|  | **Disagree** | 23 (36.5) | 20 (33.9) | 3 (16.7) |  |
| **CHW health education** | **Agree** | 34 (54) | 35 (59.3) | 14 (77.8) | .193 |
|  | **Disagree** | 29 (46) | 24 (40.7) | 4 (22.2) |  |
| **CHW health services** | **Agree** | 37 (58.7) | 38 (64.4) | 14 (77.8) | .329 |
|  | **Disagree** | 26 (41.3) | 21 (35.6) | 4 (22.2) |  |
